# Knowledge, attitude, and practice of breast self-examination is associated with general self-care and cultural factors: a study from Tamil Nadu, India

**DOI:** 10.1186/s12905-024-02981-9

**Published:** 2024-03-02

**Authors:** Bhoomika N. Jadhav, E.P. Abdul Azeez, Manoj Mathew, A.P. Senthil Kumar, M.R. Snegha, G. Yuvashree, S.N. Mangalagowri

**Affiliations:** 1 School of Social Sciences and Languages, Vellore Institute of Technology (VIT), Vellore, Tamil Nadu 632014 India; 2 Department of Social Work, Kalinga University, Raipur, India; 3 School of Social Work, University of Jigjiga, Jigjiga, Ethiopia

**Keywords:** KAP, Breast self-examination, Self-care, Breast cancer, Cultural factors

## Abstract

**Aim:**

Breast cancer is the most prevalent type of cancer among women. One form of care related to early detection of breast cancer is breast self-examination (BSE). However, evidence on knowledge, attitude, and practice (KAP) of BSE and its determining factors are minuscule in an Indian context. Therefore, the present study primarily examined the prevalence of KAP of BSE. Further, its association with general self-care and cultural factors was determined.

**Methods:**

This cross-sectional study obtained data from 412 women (Mn age = 26.63) from two rural localities of Vellore district, Tamil Nadu, India. Self-reported questionnaires of KAP of BSE, self-care, and cultural factors were applied. Statistical analyses include independent sample t-test and binomial logistic regression.

**Results:**

The majority of the sample had inadequate knowledge (58%), unfavourable attitudes (73.8%), and poor practice (89.6%) of BSE. The general self-care among the sample was moderate. Self-care was found to be a significant predictor of knowledge (b = 0.07, *p* < .05) and attitude (b = 0.092, *p* < .05) toward BSE. Shyness was identified as a negative predictor of KAP. Discouraged breast health discussions predicted inadequate knowledge, and not being educated by family/friends had a negative impact on knowledge and practice. A preference for same-gender physicians led to an unfavourable attitude toward BSE.

**Conclusion:**

The observed negative trends in KAP of BSE are concerning. The results imply that girls and women should be educated and encouraged to practice BSE and promote self-care behaviours. At the same time, efforts to reduce cultural barriers may be helpful to promote the KAP of BSE.

## Introduction

Breast cancer is a leading diagnosis among women worldwide. The 5-year estimated prevalence of breast cancer accounted for 7,790,717 (30.3%) cases, according to reports from the Global Cancer Observatory (GCO) for females of all ages [1], with 6,85,000 deaths caused in 2020 [2]. In India, breast cancer ranked fourth on the list during the 1990s and has now become the most prevalent of all cancers among women [3, 4]. The reports from national cancer registries showed the highest incidence for Delhi (41/100,000 women), followed by Chennai, Tamil Nadu (37.9/100,000 women) [5]. Breast cancer is metastatic in nature. Hence, early detection of abnormal cells may have a high probability of better prognosis and survival [6]. In this view, several screening approaches, such as mammography and magnetic resonance imaging (MRI), have gained significance, especially in asymptomatic populations [7]. However, due to the insensitivity of mammography in subpopulations of women [8], the high cost of MRI, and the lack of access to equipped healthcare centres, medical-based screening methods are sidelined in low and middle-income countries [9]. Nevertheless, adequate knowledge, a favourable attitude, and good practice toward screening for breast cancer are essential, and evidence exists otherwise [10, 11].

Breast self-examination (BSE) is a monthly procedure followed at the individual level to examine and detect breast abnormalities [12]. This non-invasive procedure has been a key method in the detection of breast cancer, and a study from Nigeria found that every nine out of ten women find the lump themselves, though eight of those are noncancerous [13]. Similarly, another study from the US indicated that a significant proportion of the women sample (43%) detected breast cancer through self-examination or accidently [14]. In addition, the American Cancer Society [15] recommends educating and teaching BSE from high school onwards, considering its significance to women’s self-care regime. Several studies have observed that BSE’s knowledge, attitude, and practice (KAP) were inadequate [16–18]. Women in metropolitan cities are knowledgeable about BSE but are unaware of performing it appropriately [19], while women from rural areas have poor KAP [20]. Studies from different countries showed that most women are diagnosed during the later stages of cancer and are the ones who did not practice BSE [19, 21, 22].

General self-care is an intense process by which individuals carry out to understand and know their bodies and how they will respond to health deviations [23]. A wide interest in self-care activities performed by the general public is identified as a promotive factor for well-being [24] and, hence, is being encouraged. Self-care among women as a subject has been popular in the literature. However, self-care practices are challenging for women amidst their commitments to providing family care, caring for children, meeting daily demands, dealing with illnesses, and negotiating work or occupational needs [25]. Despite this fact, women are also more inclined towards performing preventive self-care routines [26]. However, when the subject shifts from general self-care to breast self-examination practices, there is a lower prevalence, especially among low- and middle-income countries [18, 27].

Moreover, these self-care behaviours are generally performed by oneself without the assistance of healthcare specialists, qualifying to be called an important form of early care [28]. This further bears similarity with the practice of BSE, yet is overlooked and neglected in the normal self-care routine among women. Not performing BSE is attributed to a lack of awareness regarding BSE, a low level of education, and fear of the disease among many [29–31]. Health literacy is another variable determining whether women engage in BSE [32]. Socio-demographic aspects such as education and domicile also relate to BSE [30]. In addition, culture was found to influence self-care behaviours in general [33]. In a sample of Chinese-American older women, cultural barriers were documented for breast cancer screening [34, 35].

In a recent systematic review, BSE was not listed as a recommended guideline for screening breast cancer [36]. However, BSE is performed to be aware of the normality of breasts and to recognize changes from unexplained causes. Moreover, in contrast, clinical methods like mammography and MRI detect signs of cancer even before they can be felt overtly by the individual. In this view, BSE only helps notice abnormalities that may or may not be cancerous. Hence, BSE is essentially an aspect of a bodily self-care regime. BSE cannot be called a screening method because ‘not feeling abnormalities or lumps’ does not guarantee ‘no risk or signs of breast cancer’. On the other hand, not knowing and performing BSE as part of self-care may also be harmful in a way that women may fail to notice signs potentially indicative of risks. In addition, the significance of such practices increases when the primary health centres have poor medical infrastructure, especially in low-resource countries. Therefore, the present study supports BSE as a form of early self-care rather than a screening method for breast cancer. In this context, the present study aimed to determine the KAP of BSE among women from Vellore, Tamil Nadu, India. Further, we examined the association of KAP with the general self-care and cultural factors.

## Methods

### Research design and procedure

A cross-sectional study was undertaken in two rural localities adjacent to Vellore City, Tamil Nadu, India, during August and September 2023. A cross-sectional design was ideal since the present study aimed to explore the prevalence and determinants of KAP of BSE. Cross-sectional studies are suitable for understanding prevalence and generating preliminary evidence [37]. Women aged 18 years and above (*N* = 412; Mn age 26.63 years) were recruited for this study using a systematic sampling strategy. The sample size was determined at a 95% confidence interval (*p* = .05), a 5% margin of error for an infinite population proportion (50%). The required sample size was 385. However, we collected data from 412 participants. The data from individuals with an existing cancer diagnosis of any type would be inappropriate and, hence, were excluded. Most participants (89%) used a self-administered questionnaire, while the research team assisted the remaining in filling the questionnaire. All the questionnaires were used only in their original English form. This included participant informed consent, socio-demographic information, self-care inventory, questions on cultural factors, and a questionnaire on KAP of BSE.

### Measures

#### Self-care

In this study, we have used the Self-Care Inventory (SCI), *a self-reported* instrument to assess self-care among adults, containing 20 items [38]. The subscales include maintenance, monitoring, and management concerning self-care, with reliability coefficients equal to .85, .88, and .88, respectively. The inventory maintained a 5-point Likert scale ranging from ‘never-1’ to ‘always-5’. A cumulative score on this measure ranges from 20 (low self-care behaviours) to 100 (high self-care behaviours) with strongly established construct validity.

#### Cultural factors

Cultural factors included aspects of 1) being shy, 2) not being encouraged to have conversations regarding their private parts (breasts in this study), 3) not being exposed to discussions on breast or reproductive health and abnormalities, and 4) the preference for a female physician. The responses to these items were dichotomous and recorded as yes or no. Every response marked as yes would qualify for the presence of cultural influence/belief.

#### Knowledge, attitude, and practice of BSE

To assess KAP towards BSE, a structured questionnaire consisting of 35 items was employed as used in previous studies [11, 39]. There were 15 items assessing knowledge regarding BSE and signs of breast cancer. At the same time, 13 items were for attitude and 7 items for practice of BSE. Categorical responses (True/false/don’t know) for knowledge and a 5-point Likert scale for attitude (Strongly agree to strongly not agree) and practice items (Never to always) were applied.

The scoring was based on the following criteria: ‘2’ points were given for a correct response, ‘1’ points for ‘don’t know’, and ‘0’ marks for incorrect answers for all positive knowledge-related items and the reverse for negative knowledge items. For positive attitude-related items, it started from ‘4’ for strongly agree to ‘0’ for strongly not agree, while for negative attitude-related items, the scoring was reversed. Similarly, the points ranged from ‘0’ for ‘never’ to ‘4’ for ‘always’ for good practice and vice versa for poor practice items.

A total score was obtained for each domain and categorized into adequate and inadequate knowledge, favourable and unfavourable attitude, and good and poor practice. The categorization was based on 70% cut-off from the total of each domain [40].

### Data analysis

Statistical analyses involved descriptive and inferential statistical tests and were carried out using SPSS software (Version 25). Pearson product-moment correlation determined the association between study variables. An independent samples t-test was applied to examine significant differences in KAP based on marital status. Binomial logistic regression examined the effects of self-care and cultural beliefs on the KAP of BSE.

### Ethical consideration

All participants were provided with a research information sheet. They were encouraged to ask questions about the study and their participation, which were answered satisfactorily. Informed consent was obtained in written form. Participants were informed that they could withdraw from participating at any given time without providing any reasons. Demographic identifiers such as name and residential address were not obtained to maintain the anonymity of the participants, while their responses were kept confidential throughout the study. All procedures performed in this study involving human participants followed Helsinki’s declaration.

## Results

The socio-demographic information of 412 (Mn age = 26.63 years) participants is presented in Table 1. All women were aged 18 years and older. Over half of the participants (56.31%) were graduates. More than 50% of the women were unmarried (*n* = 251). The percentage of women aware of breast cancer was 86.2%, and 63.3% of them were aware of screening for breast cancer.

**Table 1:** Demographic information of the sample (*N* = 412)

Variables	Categories	N	Percentage (%)
Age	18–30 31–46	286 126	69.42 30.58
Education	Secondary Higher Secondary Graduation and above	44 136 232	10.68 33.01 56.31
Marital status	Married Unmarried	161 251	39.1 60.9
Awareness about Breast Cancer	Yes No	355 57	86.2 13.8
Awareness of screening for breast cancer	Yes No	261 151	63.3 36.7

Table 2 represents the study variables and their composition in the sample. The majority of women demonstrated inadequate knowledge (*n* = 239; 58%), unfavourable attitudes (*n* = 304; 73.8%), and poor practice (*n* = 369; 89.6%) of BSE. The mean score for self-care behaviours among the study sample was 71.28, which was moderate in level—similarly, more than 50% of the women agreed with all four cultural factors.

**Table 2:** Study variables and their composition in the sample

Variable	Category	N (%)
Knowledge of BSE	Adequate Inadequate	173 (42.0) 239 (58.0)
Attitude toward BSE	Favourable Unfavorable	108 (26.2) 304 (73.8)
Practice of BSE	Good Poor	43 (10.4) 369 (89.6)
Shyness	Yes No	220 (53.4) 192 (46.6)
Discourage breast health discussion	Yes No	252 (61.2) 160 (38.8)
Not educated by family/friends	Yes No	234 (56.8) 178 (43.2)
Physician’s gender	Yes No	284 (68.9) 128 (31.1)
	**Mean**	**SD**
Self-care	71.28	12.89

*Notes* Descriptive statistics

The independent samples t-test showed a significant difference based on marital status for Knowledge (t = 2.11, *p* < .035), attitude (t = 2.02, *p* < .044) and practice (t = 5.43, *p* < .001) of BSE, with unmarried women having a higher mean score indicating favourable trends (Table 3).

**Table 3:** Comparison of knowledge, attitude, and practice of BSE across participants’ marital status

	Marital status	N	M (SD)	df	MD	t	p
Knowledge	M UM	161 251	20.02 (3.85) 20.86 (4.05)	410	− 0.85	2.11*	0.035
Attitude	M UM	161 251	31.50 (6.97) 32.84 (6.33)	410	-1.34	2.02*	0.044
Practice	M UM	161 251	11.14 (5.71) 14.19 (5.30)	410	-3.04	5.43*	0.001

*Notes*: Independent samples t-test; *p < .05; M-married; UM-unmarried

Results from Pearson product-moment correlation of the interest variables are displayed in Table 4. Age was negatively associated with knowledge, attitude and practice of BSE while positively correlated with self-care and cultural factors. Years of education were positively related to knowledge, attitude, practice, self-care and negatively related to two cultural factors (discouraged breast health discussion and not educated by family/friends). Knowledge of BSE was positively associated with attitude, practice, and self-care and negatively associated with cultural factors. Attitude towards BSE showed a positive relationship with practice and self-care, whereas negative relationships with all four cultural factors. Further, the practice of BSE had a significant negative relationship with cultural factors.

**Table 4:** Correlation matrix of the study variables

	1	2	3	4	5	6	7	8	9	10
1. Age	1									
2. Education	− 0.068	1								
3. Knowledge	− 0.134*	0.292*	1							
4. Attitude	− 0.176*	0.195*	0.545*	1						
5. Practice	− 0.302*	0.144*	0.281*	0.372*	1					
6. Self-care	0.134*	0.199*	0.448*	0.339*	0.062	1				
7. Shyness	0.209*	0.010	− 0.320*	− 0.501*	− 0.518*	− 0.084	1			
8. BrHD	0.175*	− 0.132*	− 0.308*	− 0.333*	− 0.368*	− 0.190*	0.633*	1		
9. NotEdF	0.293*	− 0.186*	− 0.317*	− 0.336*	− 0.456*	− 0.131*	0.531*	0.471*	1	
10. Physician’s Gender	0.287*	0.159*	− 0.040	− 0.240*	− 0.343*	0.042	0.456*	0.283*	0.346*	1

*Notes:* * is significant at 0.01 level; BrHD- discouraged Breast health discussion; NotEdF- Not educated by family/friends

In order to understand the predictive values of self-care and cultural factors on KAP of BSE, we followed binomial logistic regression, and the obtained results are summarized in Table 5. All three statistical models with knowledge, attitude, and practice of BSE as outcome variables were adequately specified and represented by the insignificance of the p-value for the Hosmer and Lemeshow Test.

**Table 5:** Binomial logistic regression of self-care and cultural factors on KAP of BSE

		Knowledge					Attitude					Practice				
		**B**	**SE**	**Exp (b)**	**p**	**CI**	**B**	**SE**	**Exp** **(b)**	**p**	**CI**	**B**	**SE**	**Exp** **(b)**	**p**	**CI**
Constant		-4.01	0.80	0.02	0.001		-6.52	1.04	0.001	0.001		-2.01	1.04	0.13	0.053	
Self-care		0.07*	0.01	1.06	0.001	1.05–1.09	0.092*	0.01	1.10	0.001	1.07–1.13	0.01	0.013	1.01	0.376	0.99-1.04
Shyness		− 0.99*	0.44	0.43	0.003	0.19-0.71	-2.60*	0.42	0.07	0.001	0.03-0.17	-1.20*	0.52	0.30	0.020	0.11-0.83
BrHD		− 0.71*	0.41	0.35	0.016	0.28-0.88	0.70	0.36	2.01	0.053	0.99-4.08	0.49	0.42	1.64	0.235	0.73-3.69
NotEdF		− 0.71*	0.38	0.59	0.010	0.29-0.85	0.10	0.33	1.11	0.754	0.58-2.13	-1.20*	0.45	0.30	0.007	0.13-0.72
Physician’s gender		0.41	0.40	1.36	0.168	0.84-2.68	-1.07*	0.31	0.34	0.001	0.19-0.63	− 0.61	0.37	0.55	0.098	0.27-1.12
*Model Summary*																
Hosmer & Lemeshow X^2^	12.37						14.13					10.02				
Df	8						8					8				
p-Value	0.073						0.078					0.263				

*Notes:* DisBrH- Discourage breast health discussion; NotEdF- Not educated by family/friends

Self-care was a positive and significant determinant of knowledge (b = 0.07, *p* = .001). As the score on the self-care parameter increases, so does the probability of women’s knowledge of BSE. The odds ratio for self-care was 1.06, indicating that the odds of women’s knowledge of BSE change by a factor of 1.06 for each 1% increase in the level of self-care performed. Cultural factors, namely shyness (b = − 0.99, *p* = .003), being discouraged in breast health discussion (b = − 0.71, *p* = .016), and not being educated by family/friends (b = − 0.71, *p* = .010) negatively predicted knowledge of BSE. The probability of women’s knowledge of BSE decreases with shyness, not being encouraged to have open conversations about their private parts and reproductive health, and not being educated by their family/friends, as indicated by the negative slope. In addition, for each 1% increase in these cultural components, knowledge of BSE decreases by 0.43, 0.35, and 0.59, respectively.

Similarly, results for women’s attitude towards BSE were positively impacted by self-care (b = 0.092, *p* = .001) while negatively associated with two of the cultural factors, namely shyness (b = -2.60, *p* = .001) and physician’s gender (b = -1.07, *p* = .001). The slopes indicate that as involvement in self-care increases, the attitude towards BSE will be favourable. In contrast, the presence of shyness and preference for the same-gender physician will cause a less favourable attitude. The odds ratio for self-care was 1.10, meaning that the odds of women having favourable attitudes changed by a value of 1.10 for each unit increase in self-care behaviours. Furthermore, the odd ratios for shyness and attitude towards the physician’s gender were 0.07 and 0.34, respectively. For each 1% increase, having a favourable attitude decreases by a factor of 0.07 and 0.34, respectively.

In the last model, with practice as an outcome, shyness (b= -1.20, *p* = .020) and being discouraged in breast health discussions (b= -1.20, *p* = .007) were significant negative predictors. Both these parameters had an odds ratio of 0.30. Hence, for every 1% increase in shyness and being discouraged, the practice of BSE will reduce by 0.30.

## Discussion

The present study examined the prevalence of knowledge, attitude, and practice of BSE among women. Further, KAP’s association with general self-care and cultural factors was determined. This study is the first to consider the KAP of BSE with general self-care among women. The results found that more than 50% of the sample had inadequate knowledge, unfavourable attitudes, and poor practice of BSE, which is consistent with the literature [16–18]. Specifically, the result aligned with a study conducted among women in the same age range, revealing that 89% had never carried out BSE, and two-thirds had poor knowledge [41]. Similarly, in another study with women aged between 20 and 49 years, 55.3% of them had poor knowledge, and 93% did not practice BSE [42]. Unfavourable attitudes toward BSE were also prevalent in women, as witnessed by the studies conducted in Ethiopia [11] and India [43]. The samples from all these research studies, including the present study, are from rural regions where various psychosocial and demographic factors are associated with carrying out BSE. They include education, area of residence, awareness about BSE, family history with breast cancer [30], health professionals’ advice on BSE, ethnicity [42], and employment [41].

Evidence regarding the lack of awareness among women about their reproductive health, specifically breast health, is documented [44]. Women’s risk of breast cancer is associated with several aspects of their reproductive system [45]. This may also explain the lower prevalence of poor KAP of BSE. Efforts to improve the KAP of BSE are essential in the face of this major public health issue. Further, BSE’s knowledge, attitude and practice were significantly different based on women’s marital status, similar to that of Dadzi & Adam’s [18] study.

Besides this, the general self-care practices were only moderate, with the majority showing cultural beliefs inhibiting KAP of BSE. Likewise, in a study by Shiri-Mohammadabad and Afshani [46], self-care was low among women living in marginal parts of the city. The need to increase self-care behaviours across different populations of women with and without major health issues is evident from previous research [47, 48]. Also, the cultural barriers to practising self-care and breast self-examination have been prevalent in many regions, comparable with the present study [49, 50]. All the interest variables from the present study were significantly correlated. The KAP of BSE and cultural factors are significantly associated, which is also established by another study [51]. Additionally, in this study, the demographic variables, namely age and years of education, showed a negative and positive correlation with KAP of BSE, respectively, aligning with earlier findings [18].

Analyses from binomial logistic regression identified positive and negative predictors of the three dimensions of BSE. Self-care was a significant positive predictor of adequate knowledge and favourable attitude towards BSE among the women. In a study, young and middle-aged women engaged in 464 illness-related self-care behaviours [52]. Likewise, the empirical study among older adults showed that women performed self-care activities and sought professional help when self-care did not meet health needs [53]. In another research, middle-aged women were involved in multilevel self-care behaviours to meet a variety of purposes, from concerns related to health, social interaction, and developmental changes [54]. Women are continuously witnessed to engage in self-care behaviours despite many challenges of everyday life underpinning their perception that personal health is important. Hence, women committed to pursuing health through self-care are expected to have better knowledge about BSE and a positive attitude. However, the knowledge and attitude towards BSE are affected by some of the cultural factors.

Shyness had a negative impact on all three dimensions of BSE. The finding from this study is comparable to research in a sample of 415 women aged between 20 and 49 years that showed shyness and modesty were determined as the most negative behaviours of BSE [55]. Not being encouraged to have discussions about breast health also indicated inadequate knowledge. In addition, when the family members and friends did not initiate and encourage conversations related to breast health, the sample from the present study had inadequate knowledge and poor practice of BSE. An unfavourable attitude was further predicted by witnessing a preference for same-gender physicians during health check-ups. The finding was consistent with the study carried out at primary healthcare facilities in the Al Hassa region of Saudi Arabia, showing that the fear of diagnosis, unacceptability of touching the breast by others, gender of the physician, and stigma were barriers to BSE [56]. Cultural factors/barriers influencing BSE and screening behaviours for breast cancer are undeniable [57].

Furthermore, self-care did not show a predictive association with the practice of BSE among the study sample. However, cultural factors such as feeling shy and not being educated about breast health or abnormalities by family/friends were witnessed as barriers to the practice of BSE. The findings were consistent with a research synthesis from a recent review [58]. Overall, the results indicated the multidimensional feature of self-care by uniquely including BSE and examining perceived cultural barriers.

Although the results are insightful, they must be applied and understood considering some limitations. The study sample was comparatively smaller and not proportionate to the important demographic features of the population. Therefore, the representative sample may not be guaranteed of the demographic region, limiting the study results’ generalizability. Also, while analyzing the practice of BSE, features such as its timing, procedure of performing BSE, and frequency were not considered. Future studies may consider these aspects in detail to generate more insightful inferences.

## Conclusion

While the study manifested negative trends concerning knowledge, attitude, and practice of BSE, being involved in general self-care can increase the chances of developing adequate knowledge and having a favourable attitude toward BSE. At the same time, cultural influences had a negative impact on all three dimensions of BSE. The researchers from the present study believe BSE should be encouraged as part of regular and general self-care and not as a screening method for breast cancer.

Previously, breast cancer screening camps were found successful in communities with people from socially backward classes, low economic, and limited healthcare facilities [59, 60]. During such camps, women should be informed of BSE and teach them how to perform BSE using visual representations of the procedure. The advantages of BSE should be brought to women’s awareness. While developing interventions, cultural appropriateness must be ensured. Further, healthcare providers may enquire women about their general self-care routines and BSE practices during visits to clinics or hospitals and highlight their importance. Awareness programmes on BSE for girls should be incorporated in schools and higher educational institutions to encourage regular practice from a young age. Outreach programs focusing on rural localities will also help strengthen awareness about BSE.

## Data Availability

The data used for this study is available on request from the corresponding author.
